# Circulating cell‐free messenger RNA enables non‐invasive pan‐tumour monitoring of melanoma therapy independent of the mutational genotype

**DOI:** 10.1002/ctm2.1090

**Published:** 2022-11-01

**Authors:** Lea Jessica Albrecht, Anna Höwner, Klaus Griewank, Smiths S. Lueong, Nils von Neuhoff, Peter A. Horn, Antje Sucker, Annette Paschen, Elisabeth Livingstone, Selma Ugurel, Lisa Zimmer, Susanne Horn, Jens T. Siveke, Dirk Schadendorf, Renáta Váraljai, Alexander Roesch

**Affiliations:** ^1^ Department of Dermatology University Hospital of Essen West German Cancer Center University Duisburg‐Essen and the German Cancer Consortium (DKTK) Essen Germany; ^2^ Bridge Institute of Experimental Tumor Therapy West German Cancer Center University Hospital of Essen University of Duisburg‐Essen Essen Germany; ^3^ Division of Solid Tumor Translational Oncology German Cancer Consortium (DKTK Partner Site Essen) and German Cancer Research Center DKFZ Heidelberg Germany; ^4^ Department of Pediatric Hematology and Oncology Department for Pediatrics III University Hospital of Essen Essen Germany; ^5^ Institute for Transfusion Medicine University Hospital of Essen Essen Germany; ^6^ Faculty Rudolf‐Schönheimer‐Institute for Biochemistry University of Leipzig Leipzig Germany

**Keywords:** biomarker, cell‐free RNA, cfRNA, liquid biopsy, melanoma

## Abstract

**Background:**

Plasma‐derived tumour‐specific cell‐free nucleic acids are increasingly utilized as a minimally invasive, real‐time biomarker approach in many solid tumours. Circulating tumour DNA of melanoma‐specific mutations is currently the best studied liquid biopsy biomarker for melanoma. However, the combination of hotspot genetic alterations covers only around 80% of all melanoma patients. Therefore, alternative approaches are needed to enable the follow‐up of all genotypes, including wild‐type.

**Methods:**

We identified *KPNA2*, *DTL*, *BACE2* and *DTYMK* messenger RNA (mRNA) upregulated in melanoma versus nevi tissues by unsupervised data mining (*N* = 175 melanoma, *N* = 20 normal skin, *N* = 6 benign nevi) and experimentally confirmed differential mRNA expression in vitro (*N* = 18 melanoma, *N* = 8 benign nevi). Circulating cell‐free RNA (cfRNA) was analysed in 361 plasma samples (collected before and during therapy) from 100 melanoma patients and 18 healthy donors. Absolute cfRNA copies were quantified on droplet digital PCR.

**Results:**

*KPNA2*, *DTL*, *BACE2* and *DTYMK* cfRNA demonstrated high diagnostic accuracy between melanoma patients’ and healthy donors’ plasma (AUC > 86%, *p* < .0001). cfRNA copies increased proportionally with increasing tumour burden independently of demographic variables and even remained elevated in individuals with radiological absence of disease. Re‐analysis of single‐cell transcriptomes revealed a pan‐tumour origin of cfRNA, including endothelial, cancer‐associated fibroblasts, macrophages and B cells beyond melanoma cells as cellular sources. Low baseline cfRNA levels were associated with significantly longer progression‐free survival (PFS) (*KPNA2* HR = .54, *p* = .0362; *DTL* HR = .60, *p* = .0349) and overall survival (*KPNA2* HR = .52, *p* = .0237; *BACE2* HR = .55, *p* = .0419; *DTYMK* HR = .43, *p* = .0393). Lastly, we found that cfRNA copies significantly increased during therapy in non‐responders compared to responders regardless of therapy and mutational subtypes and that the increase of *KPNA2* (HR = 1.73, *p* = .0441) and *DTYMK* (HR = 1.82, *p* = .018) cfRNA during therapy was predictive of shorter PFS.

**Conclusions:**

In sum, we identified a new panel of cfRNAs for a pan‐tumour liquid biopsy approach and demonstrated its utility as a prognostic, therapy‐monitoring tool independent of the melanoma mutational genotype.

## Graphical abstract

- Biomarker candidates were identified through unsupervised data mining of melanoma versus benign nevi or healthy skin transcriptomes.
- Droplet digital PCR (ddPCR) assays were developed for circulating cell‐free RNA (cfRNA) quantification.
- *KPNA2*, *DTL*, *BACE2* and *DTYMK* cfRNA demonstrated high diagnostic accuracy in melanoma.
- High baseline cfRNA levels were associated with significantly shorter progression‐free survival.
- cfRNA copies significantly increased during therapy in non‐responders regardless of therapy and mutational subtypes.

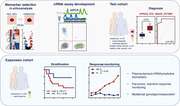

## 1 INTRODUCTION

Although recent developments have revolutionized the therapeutic landscape of melanoma by significantly improving both progression‐free survival (PFS) and overall survival (OS), the prognosis for most patients remains poor. Both early detection of recurrence or progression and selection of the right therapeutic regimen are critical factors affecting patients’ outcomes. Currently available serum biomarkers like LDH and S100B lack crucial sensitivity and specificity, and their determination is generally not recommended for detecting recurrence due to their low predictive value.^1^, ^2^ Circulating cell‐free nucleic acids are increasingly emerging as potential liquid biopsy‐based biomarkers. Recent reports highlight that the analysis of circulating cell‐free nucleic acids may provide real‐time information and allow the identification of potential new biomarkers with sufficient sensitivity to guide therapy decisions and to monitor patients with high risk for relapses such as stage III or IV patients with no evidence or minimal residual disease.^3^ Although mutant circulating tumour DNA (ctDNA)‐based liquid biopsy markers have excellent diagnostic accuracy and are able to predict therapy response, they can only be used for patients carrying melanoma‐specific mutations like *BRAF* ^V600^, *NRAS* ^Q61^ and *TERT* ^prom^ mutations.^4^, ^5^, ^6^ Besides, the combination of hotspot gene alterations only covers about 80% of all melanoma patients.^7^ Therefore, alternative approaches are needed to track all melanomas, including wild‐type tumours.

The analysis of cell‐free RNA (cfRNA) offers the possibility to detect transcriptional gene expression with high sensitivity and detectability.^8^ Due to high RNase activities in blood, the fragmentation of long RNA molecules, such as messenger RNA (mRNA), can occur^9^, ^10^, ^11^; therefore, previous studies have mainly focused on optimizing technical implementation rather than identifying potential biomarkers due to associated challenges, such as low quality and quantity of RNA in samples.^12^, ^13^, ^14^ Despite a few reports that demonstrated the use of cfRNA as a potential cancer biomarker,^15^, ^16^ currently, there are no universally applicable and reliable cfRNA biomarker candidates, and clinical application is still under investigation due to lack of standardization. Here, we identified new potential melanoma‐specific gene‐based biomarkers through *in silico* transcriptome analysis of melanoma tissues and normal skin or nevi samples and established cfRNA assays as proof‐of‐principle liquid biopsy biomarkers. We tested and validated cfRNA biomarkers across various melanoma genotypes and correlated quantitative circulating transcripts with clinicopathological variables and treatment response.

## 2 RESULTS

### 2.1 Transcriptome data mining and gene candidate identification

**FIGURE 1:**
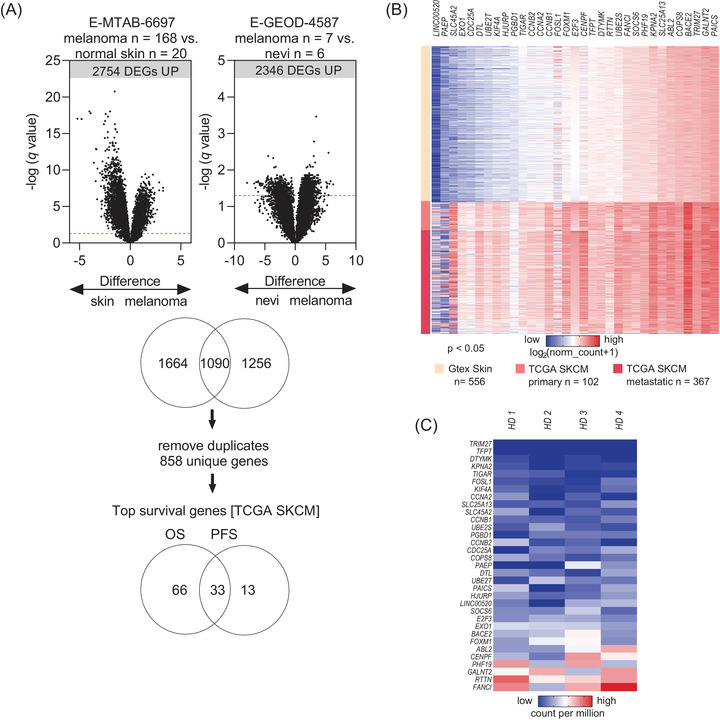
*In silico* data mining for identification of differentially expressed genes (DEGs) and DEGs selection for plasma biomarker development. (A) Processed and normalized gene expression (by array) data from two independent datasets were analysed for significant DEGs. Volcano plots show the false discovery rate (FDR) approach with *q*‐value threshold set at 5% (*q* ≤ .05). (B) Expression of the 33 genes in the validation Skin Cutaneous Melanoma cohort by The Cancer Genome Atlas (TCGA SKCM) (*N* = 102 primary and *N* = 367 metastatic melanoma tissues) and reference tissue GTEx dataset (*N* = 556 normal skin tissue samples). Heat map shows normalized gene expression (unit RSEM). (C) Expression of the 33 genes in the plasma cell‐free RNA (cfRNA) – sequencing data (*N* = 4 random healthy donors’ plasma). Heat map shows normalized gene expression (unit counts per million).

To find clinically applicable cfRNA biomarker candidates for melanoma, we started our study with an unsupervised examination of publicly available transcriptome datasets. Analysis of transcriptomic data of tumour tissue samples (*N* = 175 primary and metastatic melanoma tissues) and normal skin/nevi samples (*N* = 26) identified differentially expressed gene sets (DEGs) that were significantly upregulated in melanoma samples. Out of 1090 identified common DEGs, 858 remained that were mapped to unique genes and passed the fold change >1.5 and false discovery rate (FDR) <5% cut‐off (Figure 1A).

The Skin Cutaneous Melanoma cohort by The Cancer Genome Atlas (TCGA SKCM) is the largest untreated melanoma tissue cohort that also has curated clinical and demographic annotation. Therefore, we verified the 858 candidate DEGs on the TCGA SKCM cohort (validation dataset) and used also the largest available healthy tissue dataset (GTEx normal skin, reference dataset) for further validation. Of the 858 genes, the high expression of 99 genes was significantly associated with limited OS in the TCGA SKCM cohort. Similarly, 46 genes were markers of shorter PFS. The overlapping 33 genes were taken for further analysis. All 33 DEGs showed significantly higher expression in melanoma samples (primary and metastatic tumours) than in healthy skin tissue samples (*p* < .05, 1‐way ANOVA) (Figure 1B). Finally, to ensure the applicability of candidate genes as potential liquid biopsy cfRNA assays, we cross‐examined the expression of the 33 DEGs in a reference cfRNA‐Seq dataset derived from four random healthy donors.^16^ Genes that did not show unanimous low expression in plasma were removed from further analyses to avoid potential tissue or cell‐type specificity bias (Figure 1C). The final 13 candidates (12 protein‐coding genes, 1 long non‐coding RNA) were ranked according to highest diagnostic accuracy (TCGA SKCM vs. GTEx normal skin) and according to most significant association with OS (TCGA SKCM) (Table S1). Biomarker candidate selection and analysis processes are summarized in Figure S1.

### 2.2 cfRNA represents a potential melanoma‐specific liquid biomarker

In order to experimentally validate the gene candidates from the *in silico* screening in vitro, probe‐based assays were developed. The expression levels of the gene candidates were tested in a tissue (melanoma tissues, *N* = 18; nevi tissues, *N* = 8) and a plasma test cohort (baseline plasma samples of the same melanoma patients, *N* = 18; plasma of healthy donors, *N* = 18) using droplet digital PCR (ddPCR) (Figure S2A,B). Overall, all 13 gene candidates showed significantly higher (*p* < .05) RNA copies in melanoma tissues when compared to nevi tissues. Receiver operating characteristic (ROC) curves showed an area under the curve (AUC) >90.0% for *KPNA2*, *DTL*, *BACE2*, *DTYMK*, *E2F3* and *SLC25A13*, >80.0% for *CCNA2*, *FOXM1*, *KIF4A*, *SLC45A2*, *COPS8*, *SLC45A13* and 70.0% for *CDC25A* and *LINC00520*; all significantly discriminating melanoma from benign nevi. *ACTB* was used as endogenous control (Figure S3).

**FIGURE 2:**
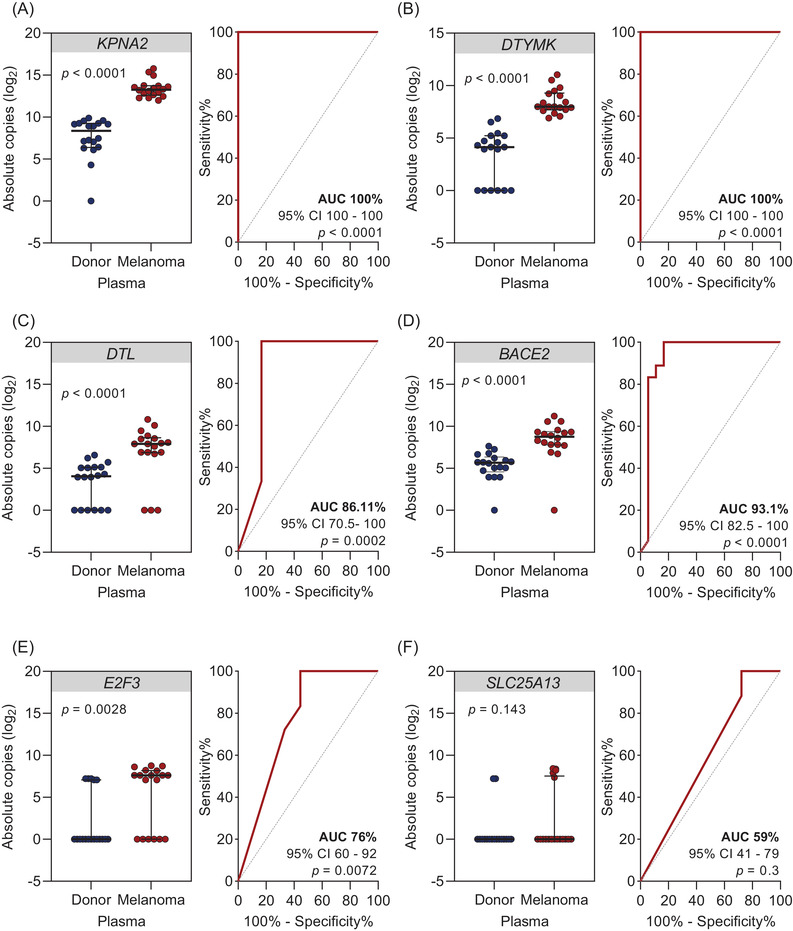
Detectable cell‐free RNA (cfRNA) levels of gene candidates in baseline plasma samples of melanoma patients versus healthy donors. (A–F) Scatter dot plots showing cfRNA levels in plasma samples of melanoma patients (stage IV *N* = 17; stage II *N* = 1) compared to healthy donors (*N* = 18) using droplet digital PCR (ddPCR). Absolute cfRNA copies and corresponding receiver operating characteristic (ROC) analysis plots of cfRNA levels of (A) *KPNA2*, (B) *DTL*, (C) *BACE2*, (D) *DTYMK*, (E) *E2F3* and (F) *SLC25A13*. Significance was assessed by Mann–Whitney *U* test. Scatter dot plots represent median values and interquartile ranges.

Next, plasma cfRNA samples from melanoma patients (*N* = 18) versus control plasma of healthy donors (*N* = 18) were used to determine gene expression levels of six gene candidates (*KPNA2*, *DTL*, *BACE2*, *DTYMK*, *E2F3* and *SLC25A13*) that showed excellent diagnostic accuracy (AUC >90.0%) in tissue (Figure 2A–F). Four of those showed reliably quantifiable cfRNA copies in plasma samples and specific expression to melanoma (*KPNA2*, *DTL*, *BACE2* and *DTYMK*) (Figure 2A–D). All four genes were significantly higher in melanoma patients’ plasma as compared to healthy donors’ plasma (*p* < .0001). In ROC AUC analysis, all four genes showed good or excellent diagnostic accuracy, with AUC 100% for *KPNA2* and *DTYMK*, 86.11% for *DTL* and 93.1% for *BACE2*.

### 2.3 *KPNA2*, *DTL*, *BACE2* and *DTYMK* cfRNAs are potential biomarkers for melanoma tumour load irrespective of the mutational genotype

To validate our findings on a larger clinical cohort, we screened our biobank for stored plasma samples of melanoma patients. In our expansion cohort, 100 melanoma patients were included (Figures S4 and S5). To exclude confounding variables and to ensure universal applicability across various melanoma subtypes, baseline cfRNA levels were analysed according to different demographic and clinicopathological subgroups. Across 64 male and 36 female patients, (median age 63 years, range 20–88 years) absolute cfRNA copies showed no significant expression differences (Figure S6A–D). Similarly, age groups (<45 years *N* = 17, 45–59 years *N* = 27, 60–74 years *N* = 36, >75 years *N* = 20) were also not significantly affected by cfRNA expression levels (Figure S6E–H). The expansion cohort included 85 cutaneous, 11 occult, 3 mucosal and 1 ocular melanoma. There were no statistically significant differences between gene expression profiles and the different histopathological variables (Figure S6I–L).

**FIGURE 3:**
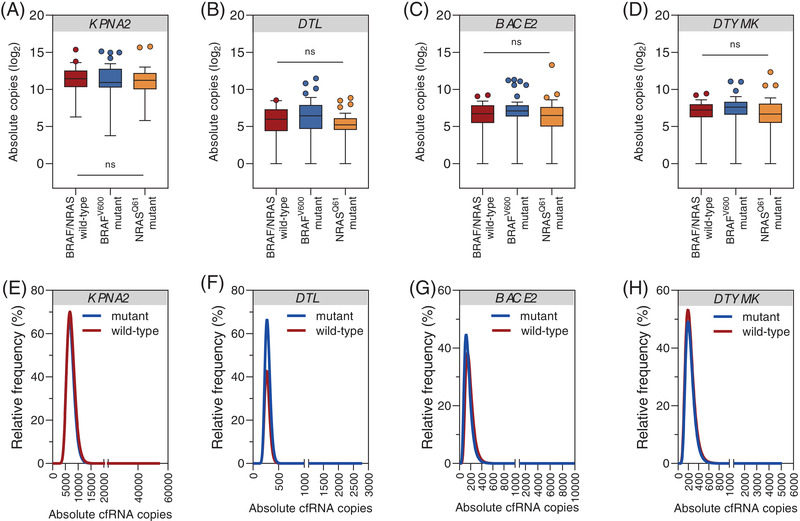
Baseline cell‐free RNA (cfRNA) levels across different mutational subtypes of melanoma. (A–D) Box and whisker plots showing detectable cfRNA levels in plasma samples of melanoma patients classified by mutational subtypes (*BRAF*/*NRAS* wild‐type ± *TERT* ^prom^ *N* = 33 vs. *BRAF* ^V600^ *N* = 41 vs. *NRAS* ^Q61^ *N* = 25). Absolute cfRNA copies of (A) *KPNA2*, (B) *DTL*, (C) *BACE2*, (D) *DTYMK*. Significance was tested by the Kruskal–Wallis test with Dunn's correction for multiple comparisons. Box and whisker plots represent median values and interquartile ranges. (E–H) Histograms showing the distribution of cfRNA levels according to absolute copy numbers between *BRAF*/*NRAS* mutant and wild‐type melanomas from non‐linear curve fitting (log–normal). Relative frequencies of (E) *KPNA2*, (F) *DTL*, (G) *BACE2*, (H) *DTYMK*.

We also analysed cfRNA copies of *KPNA2*, *DTL*, *BACE2* and *DTYMK* across different mutational genotypes of melanoma tissue (*N* = 33 *BRAF*/*NRAS* wild‐type, of those *N* = 4 positive for *TERT* ^prom^; *N* = 41 *BRAF* ^V600^ mutant and *N* = 25 *NRAS* ^Q61^ mutant melanomas). There were no significant differences in cfRNA copies among different mutational subgroups (Figure 3A–D). The relative frequency of absolute cfRNA copies followed similar distribution between mutant and wild‐type cases (Figure 3E–H). These results indicated that our cfRNA candidates could be applied as universal markers independently of tumour subtype or mutational profile.

**FIGURE 4:**
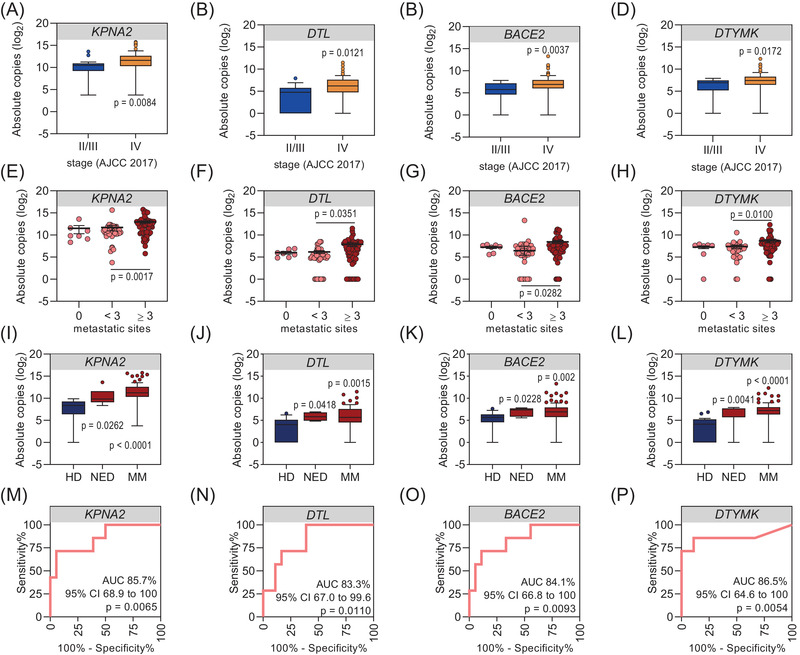
Baseline cell‐free RNA (cfRNA) copies proportionally increase with increasing tumour stage and burden and indicate the diagnostic potential for patients with no radiological evidence of disease. (A–D) Box and whisker plots showing the distribution of cfRNA levels in plasma samples of melanoma patients as classified by AJCC stage (stage II *N* = 1; stage III *N* = 16; stage IV *N* = 83). Absolute cfRNA copies of (A) *KPNA2*, (B) *DTL*, (C) *BACE2*, (D) *DTYMK*. Significance was tested by Mann–Whitney *U* test. (E–H) Scatter dot plots showing the distribution of cfRNA levels in plasma samples of melanoma patients classified according to the number of melanoma affected organs/sites (0 metastatic sites *N* = 7, <3 metastatic sites *N* = 37, ≥3 metastatic sites *N* = 56). Absolute cfRNA copies (E) *KPNA2*, (F) *DTL*, (G) *BACE2*, (H) *DTYMK*. Significance was tested by the Kruskal–Wallis test with Dunn's correction for multiple comparisons. Scatter dot plots show the mean ± SEM. (I–L) Box and whisker plots showing detectable cfRNA levels in plasma samples of melanoma patients grouped according to metastatic disease (MM, *N* = 93) and no evidence of disease (NED, *N* = 7) versus healthy donors (HD, *N* = 18). Absolute cfRNA copies of (I) *KPNA2*, (J) *DTL*, (K) *BACE2*, (L) *DTYMK*. Significance was tested by the Kruskal–Wallis test with Dunn's correction for multiple comparisons. Box and whisker plots represent median values and interquartile ranges. (M–P) Corresponding receiver operating characteristic (ROC) analysis showing HD versus NED from (I–L).

Next, we wondered whether there is a correlation between cfRNA levels and tumour burden. We grouped patients according to the AJCC stage (8^th^ edition, AJCC 2017) (stage II *N* = 1, stage III *N* = 16, stage IV *N* = 83) and the number of melanoma‐affected organ sites and analysed the distribution of cfRNA copies at baseline. Indeed, cfRNA levels significantly increased with increasing AJCC stage (*p* = .0084 for *KPNA2*, *p* = .0121 for *DTL*, *p* = .0037 for *BACE2* and *p* = .0172 for *DTYMK*) (Figure 4A–D) and M stage (*p* < .05; Figure S6M–P). For correlation of cfRNA levels with tumour burden, patients were classified according to the number of metastatic sites (0 metastatic site *N* = 7, <3 metastatic sites *N* = 37, ≥3 metastatic sites *N* = 56). Significantly higher cfRNA copies were detected in patients with ≥3 (*p* = .0017 for *KPNA2*, *p* = .0351 for *DTL*, *p* = .0282 for *BACE2* and *p* = .0100 for *DTYMK*) as compared to patients with <3 metastatic sites. Overall, cfRNA levels increased with increasing tumour burden (Figure 4E–H).

Interestingly, detectable levels of *KPNA2*, *DTL*, *BACE2* and *DTYMK* were not exclusive to plasma samples from metastatic/non‐resectable melanoma patients, as we also observed detectable cfRNA copies in melanoma patients with radiologically no evidence of disease (NED, *N* = 7, e.g., after surgical removal or complete response to drug treatment) at a similar level. Although cfRNA copies were significantly higher in metastatic patients’ (*N* = 93) versus healthy donors’ (*N* = 18) plasma (*p* < .0001 for *KPNA2* and *DTYMK*, *p* = .0015 for *DTL*, and *p* = .002 for *BACE2*); they were also higher – although to a lesser extent – in NED (*N* = 7) versus healthy donors plasma (*p* = .0262 for *KPNA2*, *p* = .0418 for *DTL*, *p* = .0228 for *BACE2* and *p* = .0041 for *DTYMK*) corresponding to high diagnostic accuracy (AUC >83%, *p* < .05) (Figure 4I–P). Moreover, increasing cfRNA copies after complete resection indicated eventual disease progression (Figure S7).

### 2.4 Prognostic value and potential therapy monitoring use of *KPNA2*, *DTL*, *BACE2* and *DTYMK* cfRNA

**FIGURE 5:**
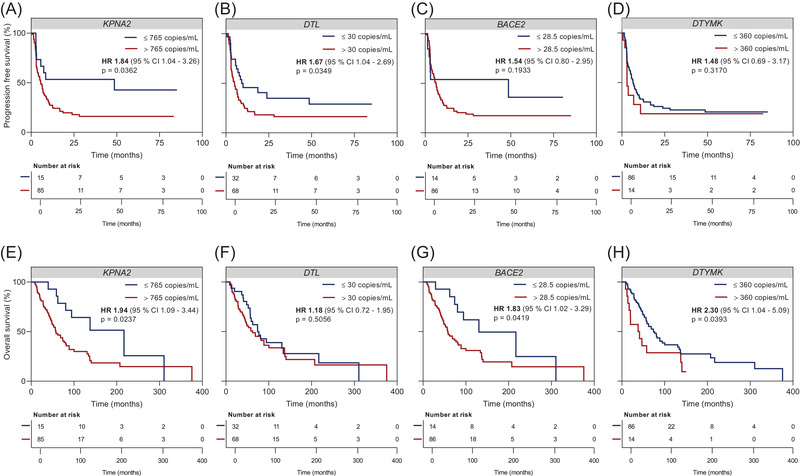
Predictive and prognostic value of baseline cell‐free RNA (cfRNA) levels. Kaplan–Meier plots of (A–D) radiologic progression‐free survival and (E–H) overall survival of melanoma patients (*N* = 100) with cfRNA copy number of (A/E) *KPNA2*, (B/F) *DTL*, (C/G) *BACE2*, (D/H) *DTYMK* classified by threshold (765 copies/ml for *KPNA2*, 30 copies/ml for DTL, 28.5 copies/ml for *BACE2*, 360 copies/ml for *DTYMK*) calculated by X‐tile software.^17^ The hazard ratio is indicated for cfRNA levels above the calculated thresholds. The *p* values were determined by the log‐rank test.

We next analysed the clinical utility of baseline cfRNA levels regarding PFS and OS. To derive a relevant cfRNA copy value threshold for *KPNA2*, *DTL*, *BACE2* and *DTYMK*, we used the X‐tile software, an outcome‐based threshold optimization tool.^17^ For *KPNA2*, a baseline cfRNA copy number above the calculated threshold at 756 copies/ml was associated with significantly shorter PFS (HR = 1.84, *p* = .0362) and OS (HR = 1.94, *p* = .0237) (Figure 5A,E). High baseline *DTL* levels (threshold at 30 copies/ml) were associated with significantly shorter PFS (HR = 1.67, *p* = .0349), but not OS (HR = 1.18, *p* = .5056) (Figure 5B,F). Although high baseline *BACE2* (threshold at 28.5 copies/ml) showed a similar trend, statistical significance was not reached for PFS (HR = 1.54, *p* = .1933), high baseline *BACE2* was predictive of significantly shorter OS (HR = 1.83, *p* = .0419) (Figure 5C,G). Finally, high baseline *DTYMK* (threshold at 360 copies/ml) showed no association with PFS (HR = 1.48, *p* = .3170) but was predictive of significantly shorter OS (HR = 2.30, *p* = .0393) (Figure 5D,H).

### 2.5 Increasing cfRNA copies reflects disease progression independently of mutational genotype or therapy type

To evaluate the potential utility of selected cfRNAs as therapy‐monitoring biomarkers, 86 patients with available baseline and longitudinal blood samples (38 responders and 43 non‐responders treated between December 2013 and May 2021) were included in the analysis (Figures S4 and S5B). Plasma cfRNA copies at four consecutive follow‐up (FU) time points (FU1: weeks 3–6, FU2: weeks 9–12, FU3: weeks 15–18, FU4: week ≥24) were compared to baseline before therapy (baseline: week 0, or up to 6 weeks before therapy start) time point.

**FIGURE 6:**
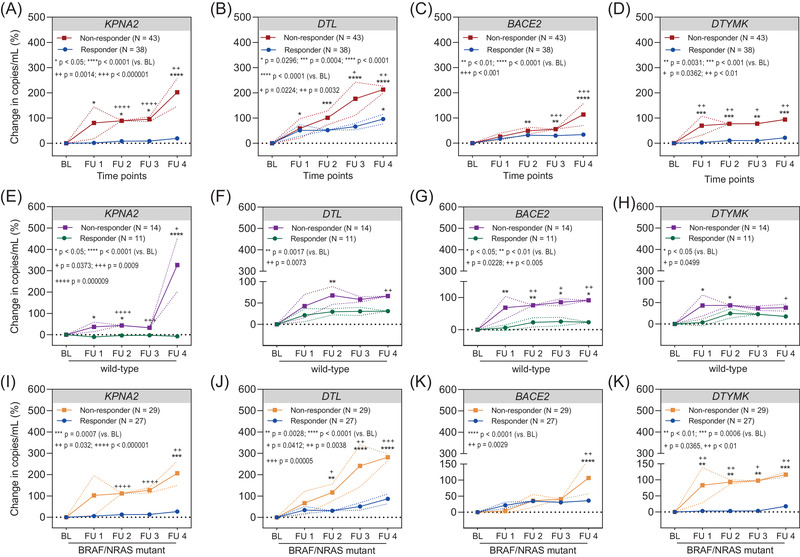
Changes in cell‐free RNA (cfRNA) levels during therapy correlate with therapy response. Changes in mean cfRNA levels of (A) *KPNA2*, (B) *DTL*, (C) *BACE2*, (D) *DTYMK* in plasma samples of melanoma patients were classified as responders (*N* = 38) and non‐responders (*N* = 43) according to radiologic response results. Changes in mean cfRNA levels: (E) *KPNA2*, (F) *DTL*, (G) *BACE2*, (H) *DTYMK* in wild‐type melanoma patients (responders *N* = 11; non‐responders *N* = 14). Changes in mean cfRNA levels of (I) *KPNA2*, (J) *DTL*, (K) *BACE2*, (L) *DTYMK* in plasma samples of *BRAF* or *NRAS* mutant melanoma (responders *N* = 27; non‐responders *N* = 29) patients. The relative change in cfRNA levels at different follow‐up (FU) time points (FU1: weeks 3–6, FU2: weeks 9–12, FU3: weeks 15–18, FU4: week ≥24) were calculated in comparison to baseline (baseline time point: week 0, or up to 6 weeks before therapy start) or preceding FU evaluation. The data represent mean ± SEM. Asterisks indicate significance of FU time point to baseline, whereas plus signs indicate significance of the same FU time point between responders and non‐responders. The *p* values for comparison of each FU time point to baseline were determined by two‐way ANOVA with Holm Sidak correction test. The *p* values for comparison of paired time points between responders and non‐responders were determined by multiple *t*‐tests.

In non‐responders, *KPNA2* cfRNA copies gradually increased during each FU time point as compared to baseline (FU1: *p* = .0304; FU2: *p* = .0300; FU3: *p* = .0419; FU4: *p* < .0001), whereas in responders no to only a modest increase was seen. More importantly, the relative *KPNA2* copy increase was significantly higher in non‐responders as compared to responders (FU2 and FU3: *p* < .000001, FU4: *p* = .0014) (Figure 6A). In non‐responders, *DTL* cfRNA copies gradually increased during each FU time point as compared to baseline (FU1: *p* = .0296, FU2: *p* = .0004; FU3 and FU4: *p* < .0001), whereas in responders an increase was seen reaching significance only at the last FU time point (FU4: *p* = .0142). Furthermore, the relative *DTL* copy increase was significantly higher in non‐responders as compared to responders (FU3: *p* = .0244, FU4: *p* = .0032) (Figure 6B). In non‐responders, *BACE2* cfRNA copies gradually increased during each FU time point as compared to baseline (FU2: *p* = .0024; FU3: *p* = .0096; FU4: *p* < .0001), whereas in responders only a slight initial increase was seen. The relative *BACE2* copy increase was also significantly higher in non‐responders as compared to responders (FU3: *p* = .0009; FU4: *p* = .0001) (Figure 6C). *DTYMK* cfRNA copies increased rapidly at the first FU time point compared to baseline and remained at a constantly elevated level in non‐responders (FU1: *p* = .0004; FU2: *p* = .0003; FU3: *p* = .0031; FU4: *p* = .0003), whereas in responders there was only a minor increase. The relative *DTYMK* copy increase was significantly higher in non‐responders as compared to responders (FU1: *p* = .0018; FU2: *p* = .0023; FU3: *p* = .0362; FU4: *p* = .0084) (Figure 6D).

To see how cfRNA levels change across different mutational subgroups, our further statistical analysis took into consideration the mutational status of melanoma patients, distinguishing between *BRAF*/*NRAS* wild‐type (*N* = 25) (Figure 6E–H) and *BRAF* or *NRAS* mutant (*N* = 56) cohorts (Figure 6I–L). Therapy responders in both cohorts (wild‐type vs. mutant) showed either no increase or an initial non‐significant increase during therapy that remained constant throughout FU time points. Non‐responders on the other hand had significantly increasing cfRNA levels during FU. More importantly, the relative cfRNA copy increase was significantly higher in non‐responders as compared to responders independently of the tumour mutational genotype (Figure 6E–L).

Finally, we wanted to see whether the different therapies induce changes in cfRNA copies. Thus, we grouped patients who received either immune checkpoint inhibition (ICI, *N* = 55) or targeted therapy (TT, *N* = 26). Overall, we observed the same kinetics for the four genes in both therapy types. Non‐responders showed statistically significantly increasing cfRNA copies during therapy, whereas responders had only a minor increase during therapy (Figure S8). However, we noticed that the increase of cfRNA copies of non‐responders was more profound starting from the first FU time point in ICI‐treated patients (Figure S8A–D). Interestingly, the cfRNA increase in TT‐treated patients seemed to follow a more subtle, gradual kinetics for all gene candidates, except for *DTL* (Figure S8E–H).

### 2.6 cfRNA copies during ICI therapy reflect gene expression changes in the tumour microenvironment

**FIGURE 7:**
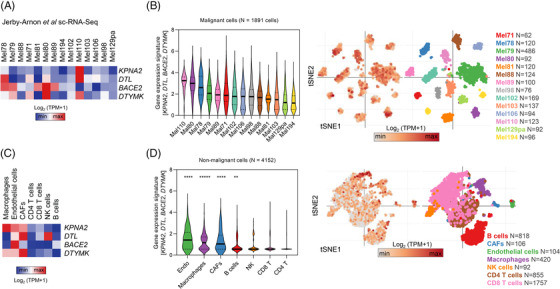
Expression of *KPNA2*, *DTL*, *BACE2* and *DTYMK* in malignant and non‐malignant cell populations. (A) Heat map showing gene expression of *KPNA2*, *DTL*, *BACE2* and *DTYMK* in malignant cells from the Jerby‐Arnon *et al*. single‐cell RNA‐Seq (sc‐RNA‐Seq) dataset.^20^ (B) Violin and tSNE‐plots showing the mean expression (*z*‐score) of *KPNA2*, *DTL*, *BACE2* and *DTYMK* in malignant cells. Only tumours with >50 total cells are shown. (C) Heat map showing gene expression of *KPNA2*, *DTL*, *BACE2* and *DTYMK* in non‐malignant cell types from the Jerby‐Arnon *et al*. sc‐RNA‐Seq data.^20^ (D) Violin and tSNE‐plots showing the mean expression (*z*‐score) of *KPNA2*, *DTL*, *BACE2* and *DTYMK* in non‐malignant cell types. Only cell types with >50 total cells are shown. Asterisks indicate *p* < .00001 from the Kruskal–Wallis test with Dunn's correction for multiple‐comparison test.

Next, we hypothesized that the different cfRNA dynamics between ICI‐ and TT‐treated patients observed previously (Figure S8) could be explained by the physiologic origin of cfRNA. Previous reports suggested that cfRNA captures transcripts that reveal cell‐ or tissue‐specific functional and genetic information.^18^, ^19^ Through the re‐analysis of single‐cell RNA‐Seq (sc‐RNA‐Seq) data derived from 31 melanoma patients,^20^ we found that *KPNA2*, *DTL*, *BACE2* and *DTYMK* are not only expressed by melanoma cells (Figure 7A,B), but also by non‐malignant cells such as endothelial cells, cancer‐associated fibroblasts (CAFs), and immune cells (Figure 7C,D). *KPNA2* and *DTYMK* were also enriched in macrophages, whereas *DTL* showed marked expression in natural killer cells (Figure 7C). Altogether, the mean expression of *KPNA2*, *DTL*, *BACE2* and *DTYMK* were significantly higher in endothelial cells, macrophages, CAFs, and B cells as compared to other non‐malignant cell types such as T cells (Figure 7D). These findings support the possibility that changes in *KPNA2*, *DTL*, *BACE2* and *DTYMK* cfRNA copies during ICI therapy reflect not only melanoma cell‐intrinsic gene expression but also strong gene expression signals from the stromal compartment.

The role of our liquid biopsy candidate biomarkers in ICI response prediction is further supported by recently published gene expression signatures.^21^ Riaz *et al*. screened transcriptome signatures of matched baseline (BL) and on‐therapy melanoma tumour biopsies of nivolumab responders and non‐responders to identify genes that are indicative of therapy response. We were intrigued to see that among the 2670 genes, which Riaz *et al*. found differentially expressed between BL and on‐therapy samples (*q* < .2),^21^ *KPNA2*, *DTL* and *BACE2* were among the top 25% hits. A more refined re‐analysis of the Riaz *et al*. dataset showed a significant increase in the expression of *KPNA2* (*p* = .0137), *DTL* (*p* < .05) and *BACE2* (*p* = .0052) in on‐treatment versus BL biopsies of nivolumab non‐responders. Although *DTYMK* was not part of the Riaz *et al*. gene signature, it was also significantly associated with progression (*p* = .0209) in on‐therapy biopsies (Figure S9A–D). Besides, when we categorized patients’ on‐therapy biopsies according to gene expression levels, we found that a high expression of each of the four genes was indicative of shorter OS (*KPNA2*: HR = 2.93, *p* = .0082; *DTL*: HR = 3.17, *p* = .0042; *BACE2*: HR = 3.70, *p* = .0415; *DTYMK*: HR = 4.80, *p* = .0005) (Figure S9E–H).

**FIGURE 8:**
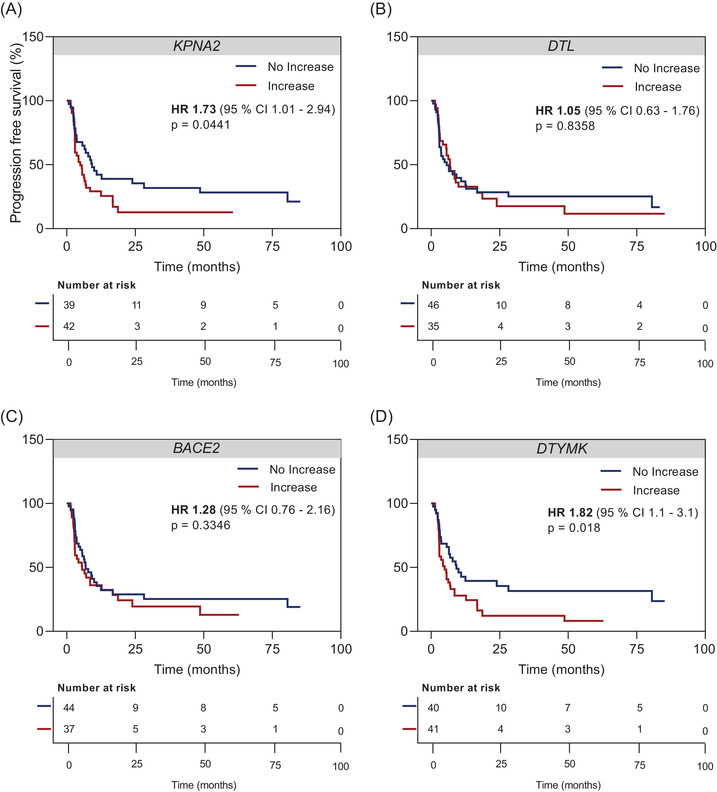
Prognostic value of cell‐free RNA (cfRNA) changes during therapy on progression‐free survival (PFS). Kaplan–Meier plots of radiologic PFS of melanoma patients (*N* = 81) according to cfRNA dynamics of (A) *KPNA2*, (B) *DTL*, (C) *BACE2*, (D) *DTYMK* during therapy. Categorization into ‘no increase’ versus ‘increase’ was based on changes of absolute copy numbers in relation to the baseline. The hazard ratio is indicated for cfRNA ‘increase’. The *p* values were determined by the log‐rank test.

Finally, as we repeatedly found significantly increasing cfRNA copies in non‐responders, we wondered if the cfRNA increase during therapy was predictive of shorter PFS. Indeed, increasing *KPNA2* copies during therapy were associated with significantly shorter PFS (HR = 1.73, *p* = .0441) (Figure 8A). *DTL* and *BACE2* increase during therapy, however, showed no significant association with PFS (*DTL*: HR = 1.05, *p* = .8358; *BACE2*: HR = 1.28, *p* = .3346), whereas *DTYMK* increase was significantly associated with shorter PFS (HR = 1.82, *p* = .018) (Figure 8B–D).

## 3 DISCUSSION

The circulating plasma transcriptome represents a valuable, currently unexplored source of potential biomarkers for cancer.^11^ The development of novel, tumour‐specific liquid biopsy assays is mostly based on the analysis of whole‐genome sequencing or whole‐exome sequencing data from tumour tissue sample banks, which can subsequently be refined into large or tailored gene panels.^22^ Several studies have shown that circulating cfRNA levels in the plasma of cancer patients are significantly higher than in plasma of healthy donors, supporting the hypothesis that in such patients the tumour is the main source of cfRNA and thus cfRNA could serve as a biomarker for disease monitoring.^23^, ^24^, ^25^ Although mRNA fragments account for up to 75% of total cfRNA, historically, many biomarker studies focus on circulating microRNAs, which account for less than 3% of the whole circulating transcriptome.^26^, ^27^

We identified gene candidates through *in silico* analyses of melanoma versus benign nevi or healthy skin transcriptomes. Our selection strategy followed the inclusion of highly abundant melanoma‐specific transcripts and the exclusion of transcripts that showed high gene expression in non‐skin tissues and blood (GTEx database, 54 different tissues from healthy individuals) to avoid confounding, that is the quantification of cfRNA signals that may originate from other non‐malignant cells or tissue sites in the body. We were able to validate that all of our shortlisted 13 gene candidates (12 mRNA, 1 non‐coding RNA) expressed significantly higher in melanoma tissues as compared to benign nevi. Furthermore, 10 of the 13 candidates were reliably detected in plasma samples of melanoma patients at significantly higher levels as compared to healthy donors’ plasma. Our final four gene candidates *KPNA2*, *DTL*, *BACE2* and *DTYMK* were selected based on the highest diagnostic accuracy (ROC AUC >90%).

*KPNA2* plays a critical role in malignant cell transformation through the transport of tumour suppressor genes, the regulation of DNA repair mechanisms and apoptosis pathways.^28^ In melanoma, *KPNA2* expression is associated with a worse prognosis, and its function in promoting proliferation, invasion and migration has been linked to NF‐κB/p65 signalling pathways.^29^ *DTYMK* is a nuclear‐encoded deoxythymidylate kinase that catalyses the phosphorylation of deoxythymidine monophosphate (dTMP) to deoxythymidine diphosphate, which is an important component of DNA synthesis.^30^ High *DTYMK* expression has been previously linked to increased melanoma cell proliferation,^31^ and shorter distant metastasis‐free survival in early stage melanoma.^32^ *DTL* is a homologue of E3 ubiquitin ligase and is involved in processes controlling cell cycle, invasion, DNA damage repair, apoptosis and DNA damage.^33^ Overexpressed *DTL* has been shown to play a role in the process of malignant transformation from nevus to melanoma tissue and correlates with poor survival rates in melanoma patients.^34^ *BACE2* is a membrane‐bound glycoprotein responsible for the proteolytic processing of soluble amyloid beta peptide. in vitro studies demonstrated that melanoma cells require amyloid beta for survival and late growth in the brain parenchyma, whereby the secretion of amyloid beta triggers local astrocytes to adopt a pro‐metastatic, anti‐inflammatory phenotype, thus suppressing intracerebral anti‐tumour immunity.^35^ Furthermore, recent reports indicate the potential role of *BACE2* in ocular melanomas as a therapeutic target.

Our results additionally showed that baseline cfRNA copies of melanoma patients did not significantly depend on age or sex. There were also no differences in histological melanoma subtypes. In line with our observations, *KPNA2*, *DTL*, *BACE2* and *DTYMK* were previously found to be associated with tumour stage also in other cancers, including hepatocellular carcinoma or glioma.^36–4^ ^7^

Our results indicate that cfRNA could complement ctDNA‐guided real‐time therapy‐monitoring approaches for patients with mutant genotypes, and more importantly, cfRNA could provide an invaluable opportunity for wild‐type patients (∼20% of all melanomas) that otherwise would be missed by classical ctDNA mutations in *BRAF/NRAS/TERT*. Although our patient cohort was not statistically powered to allow a therapy‐based subgroup comparison, the observed differences in cfRNA courses of *KPNA2*, *DTL*, *BACE2* and *DTYMK* between TT and ICI could point to a different molecular response/detectability of cfRNA depending on the therapy's mode of action. TT directly interferes with cellular processes at a molecular level,^48^ which may influence the transcription of the investigated gene candidates per se. Another explanation for the higher cfRNA levels seen in ICI non‐responders could be the cellular source of *KPNA2*, *DTL*, *BACE2* and *DTYMK* cfRNA as discussed in the following.

In contrast to ctDNA or cell‐free DNA levels reported earlier as tumour load markers,^6^, ^49^, ^50^ cfRNA copies did not decrease in responders in our patient cohorts. Moreover, we found significantly elevated cfRNA copies in individuals with a radiological absence of disease compared to healthy donors. This raises the question of whether responding patients do not become fully melanoma cell‐free at the molecular level or if cfRNA is not only a marker for tumour load but rather resembles the entire reactive ecosystem of a tumour, including stroma cells. Recent studies indeed highlighted that next to tumour‐specific transcripts, the relative contribution of other cell types that contribute to the tumour microenvironment such as CAFs, endothelial cells or immune and blood cells also represent a significant source for cfRNA.^16^, ^18^ Accordingly, our re‐analysis of published melanoma sc‐RNA‐Seq data^20^ confirmed that *KPNA2*, *DTL*, *BACE2* and *DTYMK* are not only expressed by melanoma cells, but also by endothelial cells, CAFs, B cells and macrophages at a significant level. Regarding the elevated cfRNA levels in patients with clinically NED, this could indicate that the tumour‐reactive microenvironment remains in the body beyond radiologically detectable tumour cell elimination. Looking more closely at a small subcohort of patients with NED and available FU medical history at our centre (*N* = 7), we saw that persisting cfRNA elevation could be predictive of eventual disease progression. Two patients were lost to follow‐up. Four patients relapsed during the FU, and only one patient was relapse‐free after adjuvant immunotherapy. These observations support that although all of the patients were radiologically disease‐free, baseline cfRNA copies indicated molecular progression in more than half of these patients. Whether the one patient who remains relapse‐free to date was truly disease‐free on both radiologic and molecular levels or may have extraordinarily benefited from adjuvant therapy cannot be answered retrospectively. To gain further insights into cfRNA dynamics and acquire a deeper tumour‐biological understanding, further studies with larger and more diverse patient cohorts are needed, especially regarding individual copy changes in correlation to individual responses (CR, PR or SD) and potential tumour‐independent stromal responses. In addition, clinical variables, such as overall health status and other comorbidities of the patients, need to be considered for a valid interpretation of cfRNA in the clinical decision‐making.

In summary, we have identified *KPNA2*, *DTL*, *BACE2* and *DTYMK* cfRNAs as potential pan‐tumour liquid biopsy markers for prognostic and therapy monitoring in melanoma independent of the mutational genotype. Our results warrant a further prospective validation of cfRNA as a potential biomarker for an early detection of disease progression.

## 4 MATERIALS AND METHODS

### 4.1 *In silico* data analyses of publically available datasets

In order to identify melanoma‐associated transcripts, we downloaded publicly available transcriptomic profiling data (E‐MTAB 6697,^51^ E‐GEOD 4587,^52^ discovery datasets) from ArrayExpress. The E‐MTAB 6697 meta‐dataset comprised 214 tumour tissues from basal cell carcinoma, squamous cell carcinoma, primary and metastatic melanoma samples, and normal skin. For analysis, normal skin (*N* = 20), primary and metastatic melanoma tissues (*N* = 168) were used, and other cancer entities were excluded. Similarly, from the E‐GEOD 4587 dataset, only primary or metastatic melanoma tissues (*N* = 7) and benign nevi (*N* = 6) were used. To ensure the comparability of the two datasets, processed and normalized data were analysed from the same expression‐profiling platform (A‐AFFY‐44 – Affymetrix GeneChip Human Genome U133 Plus 2.0 [HG‐U133_Plus_2]). DEGs analyses were performed using FDR applying a two‐stage step‐up multiple test correction with *q* ≤ .05 cut‐off. Genes that passed the fold change >1.5 cut‐off between melanoma versus normal skin and melanoma versus nevi were considered for further analysis. We used the skin cutaneous melanomas dataset (TCGA SKCM)^53^, ^54^ and a normal skin reference dataset (GTEx Skin) as validation cohorts. To ensure the comparability of the validation and reference tissue datasets, re‐computed expression data from the TCGA SKCM and GTEx were downloaded from the UCSC Xena database.^55^ Expressions of candidate genes in GTEx skin versus SKCM primary or metastatic melanoma were compared with one‐way ANOVA with Sidak's multiple comparisons test. AUC ROC was computed between GTEx (*N* = 556 normal skin) and TCGA SKCM (*N* = 469 melanoma tissues). Top survival‐associated genes were screened in GEPIA2,^56^ where hazard ratios and log‐rank *p* values were from univariate analysis based on median gene expression values. For final gene candidate selection, a healthy cfRNA‐Seq dataset was used that comprised four random healthy donors’ plasma. Normalized gene expression data was obtained from the authors.^16^ Genes that were absent in healthy plasma (expression value = 0) or had varying expression levels across the healthy donors were not considered. Biomarker candidate selection and analysis processes are summarized in Figure S1. sc‐RNA‐Seq data from the Jerby‐Arnon *et al*. study^20^ was visualized using the tSNE feature from the Single Cell Portal (SCP). Normalized expression data provided on the SCP was downloaded and violin plots were recreated in GraphPad Prism software (v.8.0). RNA‐Seq raw data from Riaz *et al*. (GSE91061)^21^ were downloaded and normalized by TPM (transcript per million) normalization method.

### 4.2 Plasma and tissue test cohort

Plasma samples from 18 melanoma patients (stage IV *N* = 17; stage II *N* = 1) who had prior tumour tissue excision were collected at baseline before therapy initiation. Plasma samples from standard blood preparations were collected from age and gender matched healthy donors (*N* = 18). Healthy donors had a negative melanoma history according to the pre‐blood donation questionnaires. Fresh frozen tissue samples from 18 melanomas (primary tumour *N* = 7; lymph node metastasis *N* = 5; distant organ metastasis *N* = 2 and unknown *N* = 4) were collected at baseline before therapy initiation. Common nevi tissue samples from eight donors (no previous diagnosis of melanoma) served as controls.

### 4.3 Plasma expansion cohort

Together with the test cohort, a total of 343 plasma samples from 100 melanoma patients (advanced stage III or IV: *N* = 93; fully resected high‐risk stage II/III/IV: *N* = 7) were collected. Patients received mutational testing, longitudinal blood sampling as standard of care procedures and systemic treatment if indicated as part of approval or within a clinical trial. Patients with fully resected high‐risk melanoma in the adjuvant setting received no therapy (*N* = 1) or were treated with interferon (*N* = 1), or within a clinical trial (nivolumab *N* = 2; ipilimumab plus nivolumab *N* = 2; double placebo *N* = 1). Patients in advanced/non‐resectable stages III/IV were treated with signalling TT as mono or combination therapy or in combination with chemotherapy (dabrafenib *N* = 1; binimetinib *N* = 2; dabrafenib plus trametinib *N* = 8; encorafenib plus binimetinib *N* = 5; vemurafenib plus cobimetinib *N* = 2; vemurafenib plus pexidartinib *N* = 1; trametinib plus paclitaxel *N* = 1; pazopanib plus paclitaxel *N* = 2; nintedanib plus paclitaxel *N* = 3) or ICI (pembrolizumab *N* = 18; nivolumab *N* = 20; ipilimumab *N* = 15; ipilimumab plus nivolumab *N* = 14) between December 2013 and May 2021. Plasma samples were collected prior to therapy initiation (baseline time point: week 0, or up to 6 weeks before therapy start) and at specific FU time points (first FU time point: 3–6 ± 2 weeks, second FU time point: 9–12 ± 2 weeks, third FU time point: 15–18 ± 2 weeks and long‐time FU time point: ≥24 weeks). Radiological assessments (CT and MRI) were performed as part of standard care every 12 ± 4 weeks and response assessment followed RECIST1.1 guidelines.^57^

Human biological samples and related data were provided by the Westdeutsche Biobank Essen (WBE/SCABIO, University Hospital Essen, University of Duisburg‐Essen, Essen, Germany; approval nos. 16‐7132‐BO, 17‐7729‐BO and 20‐9777‐BO). The samples were prospectively collected and archived at the local WBE/SCABIO biobank according to institutional informed consent procedures and retrospectively evaluated for this study.

### 4.4 RNA extraction

Fresh‐frozen tissues were homogenized in GentleMACS M Tubes using the GentleMACS dissociator (Miltenyi Biotec) and total RNA was isolated using the RNeasy Mini Kit (Qiagen) according to the manufacturer's instructions. Whole blood (7.5 ml) was collected in 9 ml monovettes containing EDTA (K3E, Sarstedt), and plasma was separated by centrifugation (3000 *g* for 10 min at room temperature) within 1 h of collection. The plasma phase was aliquoted into 2 ml low bind tubes and stored immediately at −80°C until isolation. Plasma samples with significant haemolysis were not included in the analysis. cfRNA isolation from 2 ml plasma was performed using the Quick‐cfRNA Serum and Plasma Kit (Zymo Research) according to the manufacturer's manual. cfRNA was eluted in 15 μl PCR grade water. RNA concentration was determined on Qubit 4.0 Fluorometer using an RNA HS (Thermo Scientific) assay kit according to manufacturer's instructions. RNA samples were stored at −80°C.

### 4.5 Assay development and transcript quantification by ddPCR

Primer and probe sequences were designed to amplify short amplicons (70–120 base pairs) using the IDT PrimerQuest Tool (Integrated DNA Technologies, Inc.) (Table S2). Dual‐labelled probes at the 5′ end with FAM or HEX fluorophores and the 3′ end with BHQ1 quencher were used. As a measure of RNA integrity and amplification efficiency, *ACTB* was used as a stably expressed endogenous control. Following standard PCR, amplicon size check was done on 2% agarose gel using an ultra‐low range DNA ladder. All sample analyses were performed in a single setup. All assays were evaluated on the QX200 Droplet Digital PCR platform (ddPCR, Bio‐Rad Laboratories). A reaction volume of 20 μl per sample was used for PCR amplification using the One‐Step RT‐ddPCR Advanced Kit for Probes (Bio‐Rad Laboratories). The final reaction mix contained 1× Supermix, 20 U/μl reverse transcriptase, 15 mM DTT, 900 nM primer pairs, 250 nM probe, 1.5 mM MgCl_2_ and .1 ng cfRNA input. A no template control was set up with PCR grade water and cell line RNA (WM3734) served as a positive control. Following PCR amplification, plates were read on the QX200 Droplet Reader, and data were analysed using QuantaSoft Software (v.1.7.4.0917, Bio‐Rad Laboratories). Detected cfRNA levels were expressed as copies per millilitre of plasma following the calculation formula established by the manufacturer. The standard deviation for each sample was calculated from the Poisson 95% confidence intervals reported in QuantaSoft. Wells containing <8000 droplets were not accepted for analysis and were repeated.

### 4.6 Statistical analysis

For statistical analysis, samples with no detectable cfRNA levels were assigned a value of 1 copy/ml. All statistical analyses and graphical presentations were performed using GraphPad Prism software (v.8.0) according to a two‐sided significance level with a 95% confidence interval, where a *p*‐value ≤.05 was considered significant. Two group comparisons (cfRNA levels compared between patient groups or clinical variables) were performed using the non‐parametric Mann–Whitney *U* test. Three or more groups’ comparisons were made by the Kruskal–Wallis test followed by Dunn's correction. Relative frequency of cfRNA copies across mutational groups was plotted as histograms using lognormal non‐linear curve fit. PFS and OS were plotted using Kaplan–Meier curves and compared using the log‐rank test. PFS was defined as the period from the date of treatment start to the date of radiographic disease progression, death or the last known FU. OS was calculated from the difference between the date of initial diagnosis and the date of death or last FU. We categorized the number of different melanoma‐involved organs according to recently published prognostically relevant divisions (<3 vs. ≥3 metastatic sites).^58^ Thresholds of cfRNA levels used for the categorization of PFS and OS were selected in X‐tile software.^17^ To evaluate the therapy monitoring potential of the four cfRNA candidates, the relative change in cfRNA copies was calculated for each patient for therapy FU time points versus baseline. Next, the mean per cent change was calculated across patients that were grouped according to radiologic response results. A cfRNA ‘increase’ or ‘no increase’ was defined as an increase or no increase in absolute copy number at the last available FU time point compared to the baseline copy number (we found that the increase at this time point was indicative of the overall cfRNA dynamics during therapy). Comparison of mean cfRNA change was performed by two‐way ANOVA with Holm Sidak's test correction, whereas comparisons of the mean cfRNA copy change of paired time points between responders and non‐responders were carried out using multiple unpaired *t*‐test.

## FUNDING INFORMATION

Brigitte and Dr. Konstanze Wegener Foundation, the Deutsche Forschungsgemeinschaft (DFG, German Research Foundation) – 405344257 (KFO 337), German Cancer Consortium (DKTK)

## CONFLICTS OF INTEREST

LJA has received honoraria form Novartis and Bristol‐Myers Squibb (BMS) and travel support from Sunpharma, Takeda and Sanofi, outside the submitted work.

AR reports non‐financial support from Amgen, non‐financial support from Roche, personal fees and non‐financial support from Merck/MSD, grants and non‐financial support from Novartis, grants and non‐financial support from BMS, and grants from Adtec outside the submitted work.

SU declares research support from BMS and Merck Serono; speakers and advisory board honoraria from Bristol Myers Squibb, Merck Sharp & Dohme, Merck Serono, Novartis and Roche and travel support from Bristol Myers Squibb, Merck Sharp & Dohme and Pierre Fabre; outside the submitted work.

JTS receives honoraria as consultant or for continuing medical education presentations from AstraZeneca, Bayer, Immunocore, Roche, Servier. His institution receives research funding from Bristol‐Myers Squibb, Celgene, Eisbach Bio, Roche/Genentech. He holds ownership and serves on the Board of Directors of Pharma15, all outside the submitted work.

AP received grants from BMS and Merck Sharp & Dohme outside the submitted work.

EL reports personal fees and other from BMS, personal fees and other from MSD, personal fees and other from Medac, personal fees and other from Novartis, personal fees from Recordati, personal fees from Sanofi, personal fees and other from Pierre Fabre, personal fees from Sunpharma, outside the submitted work.

LZ reports personal fees and other from Bristol Myers Squibb, personal fees and other from MSD, personal fees and other from Pierre Fabre, personal fees and other from Roche, other from Sanofi, other from Amgen, personal fees and other from Novartis, outside the submitted work.

DS served as a consultant and/or has received honoraria from Array, Roche, Bristol Myers Squibb, Merck Sharp & Dohme, Nektar, NeraCare, Novartis, Pierre‐Fabre, Philogen, Pfizer, Sandoz, Sunpharma and Sanofi; research funding to institution from Novartis, Amgen, Roche, MSD and Array; travel support from Merck Sharp & Dohme, Bristol Myers Squibb, Pierre‐Fabre, Sunpharma, Sanofi and Novartis, outside the submitted work.

All other authors have declared that no conflict of interest exists.

## Supporting information


**Figure S1** Flow chart summarizing biomarker candidate selection and analysis processes.

**Figure S2 Patient enrolment in the test cohort**. (A) Overview of the collected tissue and plasma samples of melanoma patients and healthy donors in the test cohort. (B) Overview of plasma (stage IV *N* = 17; stage II *N* = 1) and tissue test cohort (primary *N* = 7; lymph node *N* = 5; metastasis *N* = 2; unknown *N* = 4). The upper panels show demographic, tumour characteristics and the tissue extraction site, and the lower panels represent the mutational status of individual melanoma patients. Plasma and tissue samples were collected before therapy start (baseline time point: week 0, or up to 6 weeks before therapy start). Tissue samples from benign nevi (*N* = 8) and plasma samples (*N* = 18) from healthy individuals were included as controls.

**Figure S3 Detectable RNA levels of the 13 gene candidates in melanoma versus nevi tissue**. (A–M) Box and whisker plots showing detectable mRNA levels in melanoma tissue samples (*N* = 18) compared to nevi tissue samples (*N* = 8) using ddPCR and corresponding ROC analysis plots of mRNA levels of individual gene candidates classified by melanoma or nevi tissue. Absolute mRNA copies and corresponding ROC analysis of (A) *KPNA2*, (B) *DTL*, (C) *BACE2*, (D) *DTYMK*, (E) *CCNA2*, (F) *LINC00520*, (G) *FOXM1*, (H) *KIF4A*, (I) *SLC45A2*, (J) *E2F3*, (K) *SLC45A13*, (L) *COPS8*, (M) *CDC25A* and (N) *ACTB* (endogenous control) in melanoma tissues compared to nevi. Significance was assessed by Mann–Whitney *U* test (*p* < .05). Box and whisker plots represent median values and interquartile ranges.

**Figure S4 Patient enrolment in the expansion cohort**. Overview of the enrolled patients and plasma sample collection for cfRNA baseline and monitoring analysis in expansion cohort according to the CONSORT‐Statement.^59^

**Figure S5 Basic characteristics of the melanoma expansion cohort**. Overview of patient and tumour characteristics of melanoma expansion cohort for (A) baseline analysis (*N* = 100) and (B) therapy‐monitoring analysis (*N* = 86). The upper panels show patient characteristics, the middle panels show details of disease status and the lower panels show the mutational status. Plasma samples were collected before therapy start (baseline time point: week 0, or up to 6 weeks before therapy start) and at different follow‐up (FU) time points (FU1: 3–6 ± 2 weeks, FU2: 9–12 ± 2 weeks, FU3: 15–18 ± 2 weeks and FU4: >24 weeks).

**Figure S6 cfRNA levels at baseline according to demographic data**. (A–D) Box and whisker plots showing detectable cfRNA levels in plasma samples of melanoma patients classified by gender (male, *N* = 64; female, *N* = 36). Absolute cfRNA copies of (A) *KPNA2*, (B) *DTL*, (C) *BACE2*, (D) *DTYMK*. Significance was assessed by Mann–Whitney *U* test. (E–H) Box and whisker plots showing detectable cfRNA levels in plasma samples of melanoma patients classified by age groups (<45 years, *N* = 17; 45–59 years, *N* = 27; 60–74 years, *N* = 36; >75 years, *N* = 20). Absolute cfRNA copies of (E) *KPNA2*, (F) *DTL*, (G) *BACE2*, (H) *DTYMK*. (I–L) Box and whisker plots showing detectable cfRNA levels in plasma samples of melanoma patients classified by histological subtypes (cutaneous *N* = 85 vs. occult *N* = 11 vs. ocular *N* = 1, mucosal *N* = 3). (I) *KPNA2*, (J) *DTL*, (K) *BACE2*, (L) *DTYMK*; (M–P) scattered dot plots showing the absolute cfRNA copies of (M) *KPNA2*, (N) *DTL*, (O) *BACE2*, (P) *DTYMK* according to M stage (AJCC 8th edition, M0: *N* = 17, M1a: *N* = 6, M1b: *N* = 15, M1c: *N* = 41, M1d: *N* = 21). Significance was assessed by the Kruskal–Wallis test with Dunn's correction for multiple comparisons. Box and whisker plots represent median values and interquartile ranges. Scattered dot plots show the mean ± SEM.

**Figure S7** (A) Longitudinal assessment of cfRNA copies of *KPNA2*, *DTL*, *BACE2*, *DTYMK* in patients with no evidence of disease (NED) at baseline. (B) cfRNA copies of *KPNA2*, *BACE2*, *DTL* and *DTYMK* at baseline versus at the time of relapse were plotted for patients 7, 14, 37 and 39. Paired time points were pooled and compared by the Wilcoxon‐signed rank test.

**Figure S8 Changes in cfRNA levels during therapy classified by different therapies**. Changes in mean (copies/ml) of cfRNA levels of (A/E) *KPNA2*, (B/F) *DTL*, (C/G) *BACE2*, (D/H) *DTYMK* in plasma samples of melanoma patients classified as responders (*N* = 38) and non‐responders (*N* = 43) according to radiologic response results. The relative change in cfRNA levels at different follow‐up (FU) time points (FU1: weeks 3–6, FU2: weeks 9–12, FU3: weeks 15–18, FU4: week >24) were calculated in comparison to baseline (BL) (baseline time point: week 0, or up to 6 weeks before therapy start) or preceding FU evaluation. (A–D) Immune checkpoint inhibitors (ICI) (responders *N* = 24; non‐responders *N* = 31) and (E–H) targeted therapy (TT) (responders *N* = 14; non‐responders *N* = 12). The data represent mean ± SEM. Asterisks indicate significance of FU time point to baseline, whereas plus signs indicate significance of the same FU time point between responders and non‐responders. The *p* values for comparison of each FU time point to baseline were determined by two‐way ANOVA with Holm Sidak correction test. The *p* values for comparison of paired time points between responders and non‐responders were determined by a *t*‐test.

**Figure S9 Validation of therapy predictive and prognostic relevance of KPNA2, DTL, BACE2 and DTYMK**. (A–D) Nivolumab‐treated tumours with increasing gene expression of *KPNA2*, *DTL*, *BACE2* and *DTYMK* are associated with progression. Scattered dot plots showing the fold change in expression of (A) *KPNA2*, (B) *DTL*, (C) *BACE2* and (D) *DTYMK* in nivolumab‐treated tumours (on‐treatment vs. baseline) in the Riaz *et al*. dataset.^21^ The *p* values are from the Kruskal–Wallis test with Dunn's correction for multiple comparisons test. Scattered dot plots show the mean ± SEM. RECIST classifications: CR = complete response, PR = partial response, SD = stable disease, PD = progressive disease. (E–H) Kaplan–Meier plots of overall survival of nivolumab‐treated melanoma patients (*N* = 36) in the Riaz *et al*. dataset^21^ according to on‐treatment tumour gene expression profiles of (E) *KPNA2*, (F) *DTL*, (G) *BACE2*, (H) *DTYMK*. Categorization into ‘low’ versus ‘high’ was based on X‐tile software.^17^ The hazard ratio is indicated for the ‘high’ category. The *p* values were determined by the log‐rank test.

**Table S1** Final list of differentially expressed genes (DEGs) for cfRNA assay development ranked according to diagnostic accuracy in TCGA SKCM/GTEx normal skin and association with overall survival in TCGA SKCM.
**Table S2** Primer and probe sequences
