# Supplementary figures and images for: Circulating cell‐free messenger RNA enables non‐invasive pan‐tumour monitoring of melanoma therapy independent of the mutational genotype
Source: Clin Transl Med. 2022 Nov 1;12(11):e1090. doi: 10.1002/ctm2.1090 (PMC9626658; doi:10.1002/ctm2.1090)

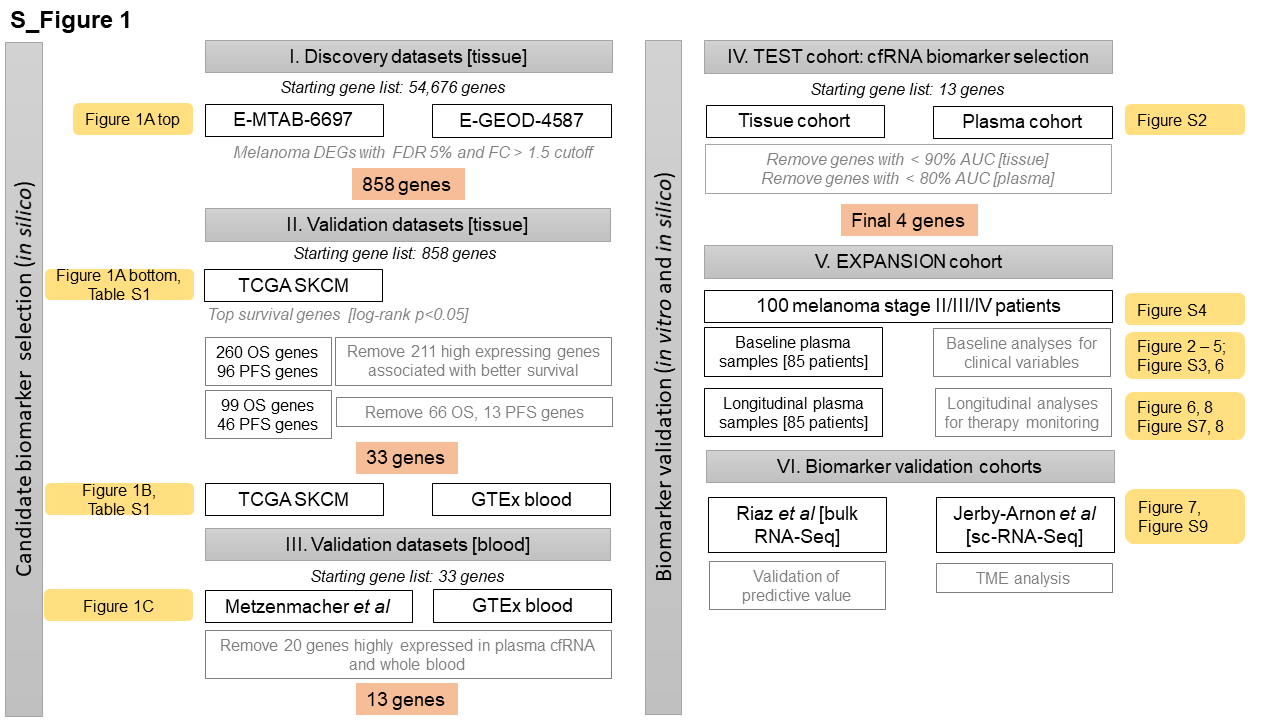

Supplement: Supplementary file 1 — Figure S1 Flow chart summarizing biomarker candidate selection and analysis processes. [file CTM2-12-e1090-s002.png]

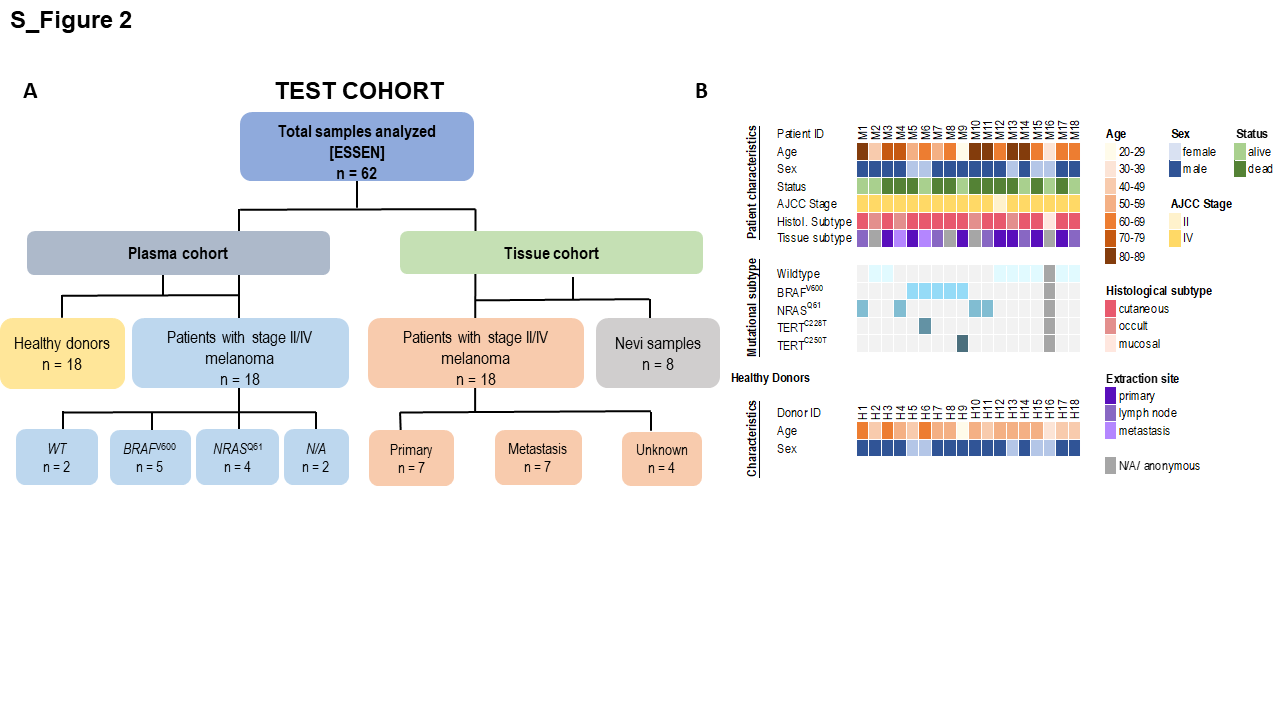

Supplement: Supplementary file 2 — Figure S2 Patient enrolment in the test cohort. (A) Overview of the collected tissue and plasma samples of melanoma patients and healthy donors in the test cohort. (B) Overview of plasma (stage IV N = 17; stage II N = 1) and tissue test cohort (primary N = 7; lymph node N = 5; metastasis N = 2; unknown N = 4). The upper panels show demographic, tumour characteristics and the tissue extraction site, and the lower panels represent the mutational status of individual melanoma patients. Plasma and tissue samples were collected before therapy start (baseline time point: week 0, or up to 6 weeks before therapy start). Tissue samples from benign nevi (N = 8) and plasma samples (N = 18) from healthy individuals were included as controls. [file CTM2-12-e1090-s010.png]

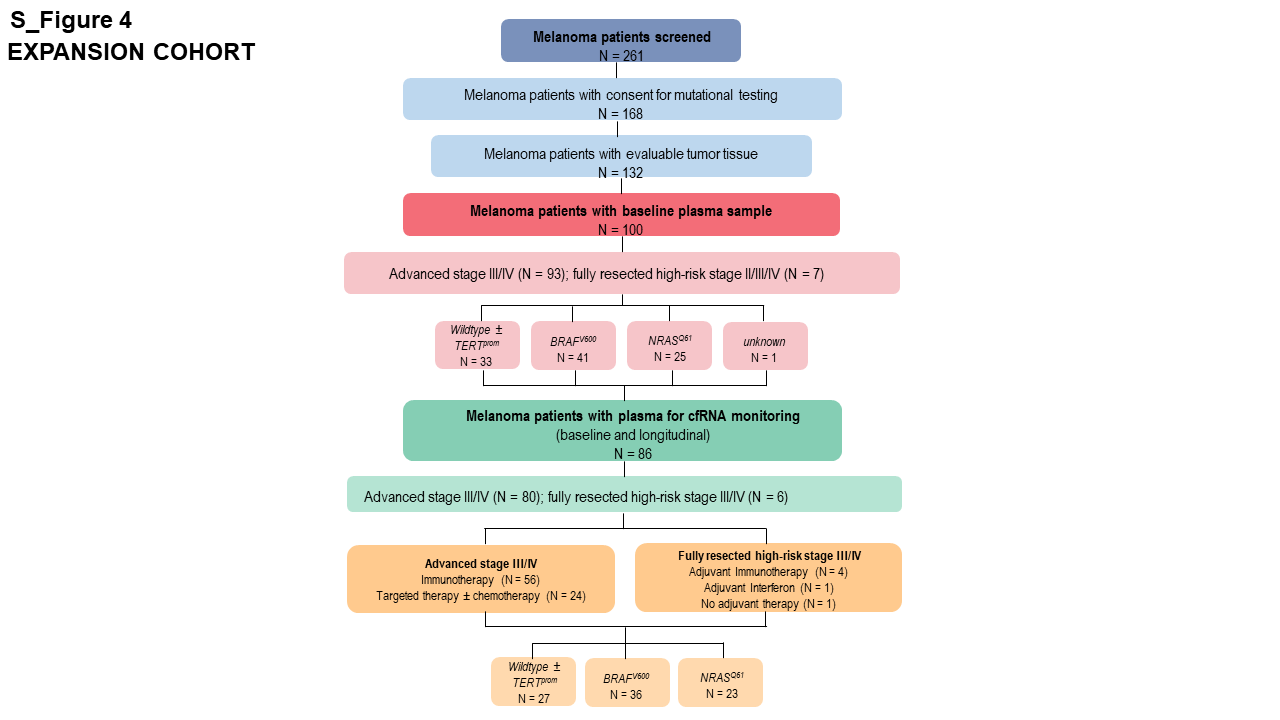

Supplement: Supplementary file 4 — Figure S4 Patient enrolment in the expansion cohort. Overview of the enrolled patients and plasma sample collection for cfRNA baseline and monitoring analysis in expansion cohort according to the CONSORT‐Statement. 59 [file CTM2-12-e1090-s001.png]

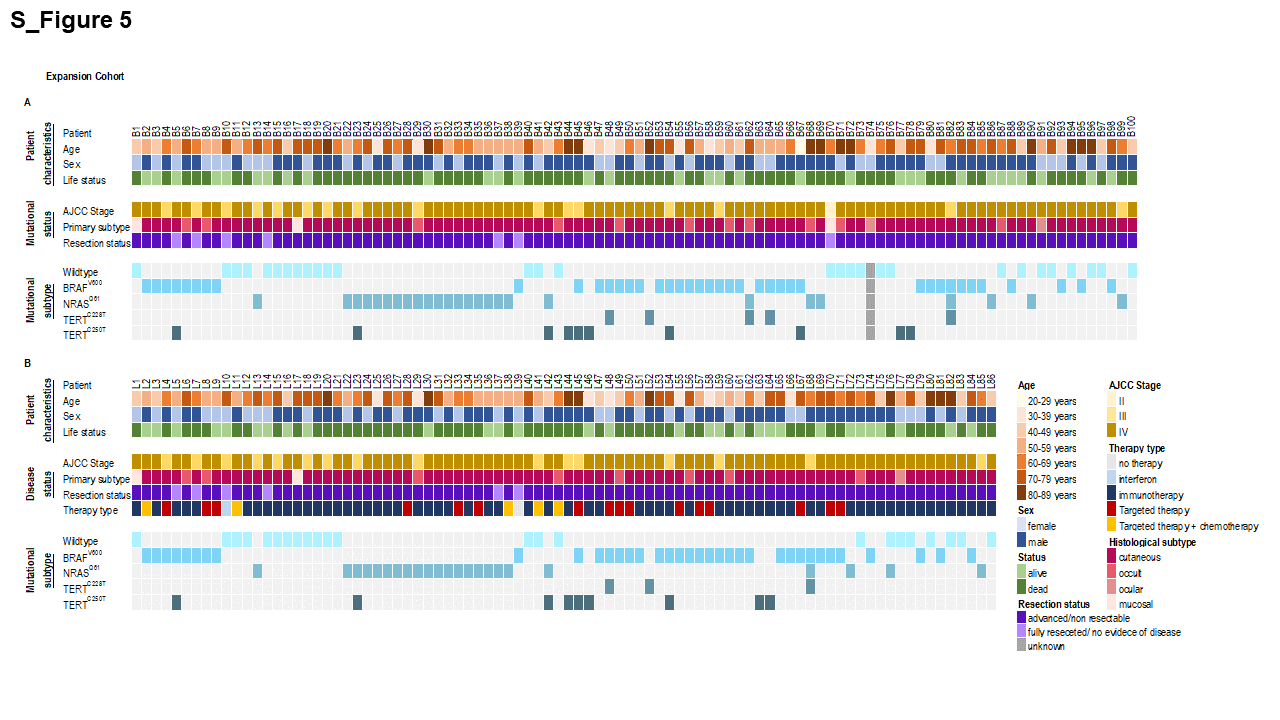

Supplement: Supplementary file 5 — Figure S5 Basic characteristics of the melanoma expansion cohort. Overview of patient and tumour characteristics of melanoma expansion cohort for (A) baseline analysis (N = 100) and (B) therapy‐monitoring analysis (N = 86). The upper panels show patient characteristics, the middle panels show details of disease status and the lower panels show the mutational status. Plasma samples were collected before therapy start (baseline time point: week 0, or up to 6 weeks before therapy start) and at different follow‐up (FU) time points (FU1: 3–6 ± 2 weeks, FU2: 9–12 ± 2 weeks, FU3: 15–18 ± 2 weeks and FU4: >24 weeks). [file CTM2-12-e1090-s003.png]
