# Supplementary material for: Circulating cell‐free messenger RNA enables non‐invasive pan‐tumour monitoring of melanoma therapy independent of the mutational genotype
Source: Clin Transl Med. 2022 Nov 1;12(11):e1090. doi: 10.1002/ctm2.1090 (PMC9626658; doi:10.1002/ctm2.1090)
Supplement: Supplementary file 10 — Table S1 Final list of differentially expressed genes (DEGs) for cfRNA assay development ranked according to diagnostic accuracy in TCGA SKCM/GTEx normal skin and association with overall survival in TCGA SKCM. Table S2 Primer and probe sequences [file CTM2-12-e1090-s005.docx]

**Supplementary Material**

**Circulating cell-free messenger RNA enables non-invasive pan-tumor monitoring of melanoma therapy independent of the mutational genotype**

Lea Jessica Albrecht^1^, Anna Höwner^1^, Klaus Griewank^1^, Smiths S. Lueong^2,3^, Nils von Neuhoff^4^, Peter A. Horn^5^, Antje Sucker^1^, Annette Paschen^1^, Elisabeth Livingstone^1^, Selma Ugurel^1^, Lisa Zimmer^1^, Susanne Horn^1,6^, Jens T. Siveke^2,3^, Dirk Schadendorf^1^, Renáta Váraljai^1, *^, Alexander Roesch^1, *^

Supplementary Table 1

Supplementary Table 2

Supplementary Figure Legends

References

**Supplementary Table 1:** Final list of differentially expressed genes (DEGs) for cfRNA assay development ranked according to diagnostic accuracy in TCGA SKCM/GTEx normal skin and association with overall survival in TCGA SKCM.

| **Gene** | **ROC AUC (%) TCGA SKCM (n=469) vs. GTEx normal skin (n=556)** | **HR for death (high expression)** | **95% CI for HR** | **Log-rank p (OS)** |
| --- | --- | --- | --- | --- |
| *KPNA2* | 99.48 | 1.60 | 1.23 to 2.01 | 0.0004 |
| *DTL* | 99.29 | 1.85 | 1.42 to 2.41 | < 0.0001 |
| *E2F3* | 99.07 | 1.59 | 1.22 to 2.07 | 0.0006 |
| *CDC25A* | 98.93 | 1.65 | 1.26 to 2.15 | 0.0002 |
| *FOXM1* | 98.91 | 1.75 | 1.34 to 2.29 | < 0.0001 |
| *KIF4A* | 98.76 | 1.36 | 1.05 to 1.78 | 0.0221 |
| *BACE2* | 98.32 | 1.66 | 1.27 to 2.17 | 0.0001 |
| *DTYMK* | 97.15 | 1.35 | 1.04 to 1.77 | 0.0235 |
| *CCNA2* | 94.24 | 1.45 | 1.11 to 1.89 | 0.006 |
| *COPS8* | 93.24 | 1.37 | 1.05 to 1.78 | 0.0166 |
| *SLC25A13* | 93.09 | 1.66 | 1.27 to 2.17 | 0.0001 |
| *SLC45A2* | 91.93 | 1.43 | 1.10 to 1.88 | 0.0063 |
| *LINC00520* | 90.29 | 1.45 | 1.11 to 1.89 | 0.0053 |

ROC: receiver operating characteristic curves; AUC: area under the curve; HR: hazard ratio; OS: overall survival

**Supplementary Table 2:** Primer and probe sequences

| **Gene** | **Size (bp)** | **Forward sequence** | **Reverse sequence** | **Probe sequence** | **5' -> 3' label** |
| --- | --- | --- | --- | --- | --- |
| *ACTB* (ctl) | 96 | GGATCAGCAAGCAGGAGTATG | AGAAAGGGTGTAACGCAACTAA | TCGTCCACCGCAAATGCTTCTAGG | HEX - BHQ1 |
| *KPNA2* | 84 | GCTTGTCCTCTGACTAGGTTTC | AGGGTGGACTTGAATGTTTATTTAC | TGTGGAATTTCCTATCTTGCAGCATCCT | FAM - BHQ1 |
| *DTYMK* | 76 | TTGGCAGTGTGGACATGAG | ATACAACAGAGTGCCATCAGG | ATGGAGCAGTCTCCTGCCCTCT | FAM - BHQ1 |
| *KIF4A* | 99 | CACCTACTGAAGAGAGAACCAAC | AGCCTGAACTGCTCACAAATA | ACTCATCAGGAACCAGTCCTCAGTCT | FAM - BHQ1 |
| *COPS8* | 98 | GCTAATAGTTCCAGGGCAGTAAG | CCCTTTGACAGAATCCCAAGT | ACAAGATGTCCAGGATTCAAGGAGGC | FAM - BHQ1 |
| *CDC25A* | 91 | CATGCACCACGAGGACTTTA | GTACATCTCCCTCTTGCTCTTC | ACCTGAAGAAGTTCCGCACCAAGA | FAM - BHQ1 |
| *FOXM1* | 94 | CCTTTGCTTCCAGTTCAGACTA | GAGGGCTCTCCACTTTGATG | TCGCTAAGTGTGGCATTTCCTCCC | FAM - BHQ1 |
| *SLC25A13* | 93 | GGGAAAGGAGCAAGTCAGAATA | AGCACCATGATTGCCTTACA | CCCTCCTTGAACTAGGATTGTAGTCCCA | FAM - BHQ1 |
| *SLC45A2* | 117 | GGGTCTTTACTTCACGGGATATT | GTGCTGGACATTACACCAAAC | ATGTCTACTCCACCCTGGTCCTGT | FAM - BHQ1 |
| *CCNA2* | 77 | AGGTAAACTCAACAGAGGTTGG | CTGGAGCATTTCTCGTCTGTTA | AGTGGAAGAGGGTGGGAAGCTTAT | FAM - BHQ1 |
| *E2F3* | 90 | GCTTCGTGTGAACTCTCCTT | GAAGAAGGTAGGAAGGGACAAC | TGGAACCAGAACATCTGTCATGCAGT | FAM - BHQ1 |
| *DTL* | 90 | AAACGGAAGGCTGAGAATCC | CGGGCTTGGCAATTTCTTTC | AATTGGGTGTCTGGGATGACGGAC | FAM - BHQ1 |
| *BACE2* | 97 | ATTTCCGGGCCTTTCTCAA | TGAGCGCATAGGACACAATC | AGGATGTAGCCAGCAACTGTGTCC | FAM - BHQ1 |
| *LINC00520* | 82 | GGACTTGCAGTTGAGCTTCT | GGGACTTGTGTTACGGTCTTC | TGCCTTGGACTTCTTGTGACTTGGA | FAM - BHQ1 |

bp: base pairs, ctl: control

**Supplementary Figure legends**

**Supplementary Figure 1**: Flow chart summarizing biomarker candidate selection and analysis processes

**Supplementary Figure 2: Patient enrollment in the test cohort.**

**A.** Overview of the collected tissue and plasma samples of melanoma patients and healthy donors in the test cohort. **B.** Overview of plasma (stage IV N=17; stage II N=1) and tissue test cohort (primary N=7; lymph node N=5; metastasis N=2; unknown N=4). The upper panels show demographic, tumor characteristics, and the tissue extraction site, and the lower panels represent the mutational status of individual melanoma patients. Plasma and tissue samples were collected before therapy start (baseline time point: week 0, or up to 6 weeks before therapy start). Tissue samples from benign nevi (N=8) and plasma samples (N=18) from healthy individuals were included as controls.

**Supplementary Figure 3: Detectable RNA levels of the 13 gene candidates in melanoma versus nevi tissue.**

**A-M.** Box and whisker plots showing detectable mRNA levels in melanoma tissue samples (N=18) compared to nevi tissue samples (N=8) using ddPCR and corresponding ROC analysis plots of mRNA levels of individual gene candidates classified by melanoma or nevi tissue. Absolute mRNA copies and corresponding ROC analysis of **A.** *KPNA2* **B.** *DTL* **C.** *BACE2* **D.** *DTYMK* **E.** *CCNA2* **F.** *LINC00520* **G.** *FOXM1* **H.** *KIF4A* **I.** *SLC45A2* **J.** *E2F3* **K.** *SLC45A13* **L.** *COPS8* **M.** *CDC25A* **N.** *ACTB* (endogenous control) in melanoma tissues compared to nevi. Significance was assessed by Mann-Whitney *U* test (p<0.05). Box and whisker plots represent median values and interquartile ranges.

**Supplementary Figure 4: Patient enrollment in the expansion cohort.**

Overview of the enrolled patients and plasma sample collection for cfRNA baseline and monitoring analysis in expansion cohort according to the CONSORT-Statement ^1^.



**Supplementary Figure 5: Basic characteristics of the melanoma expansion cohort.**

Overview of patient and tumor characteristics of melanoma expansion cohort for **A.** baseline analysis (N=100) and **B.** therapy monitoring analysis (N=86). The upper panels show patient characteristics, the middle panels show details of disease status, and the lower panels show the mutational status. Plasma samples were collected before therapy start (baseline time point: week 0, or up to 6 weeks before therapy start) and at different follow-up (FU) time points (FU1: 3-6 weeks ± 2 weeks, FU2: 9-12 weeks ± 2 weeks, FU3: 15-18 weeks ± 2 weeks and FU4: > 20 weeks).

**Supplementary Figure 6: cfRNA levels at baseline according to demographic data.**

**A-D.** Box and whisker plots showing detectable cfRNA levels in plasma samples of melanoma patients classified by gender (male N=64; female N=36). Absolute cfRNA copies of **A.** *KPNA2* **B.** *DTL* **C.** *BACE2* **D.** *DTYMK*. Significance was assessed by Mann-Whitney *U* test. **E-H.** Box and whisker plots showing detectable cfRNA levels in plasma samples of melanoma patients classified by age groups (<45 years N=17, 45-59 years N=27, 60-74 years N=36, >75 years N=20). Absolute cfRNA copies of **E.** *KPNA2*. **F.** *DTL*. **G.** *BACE2*. **H.** *DTYMK*. **I-L.** Box and whisker plots showing detectable cfRNA levels in plasma samples of melanoma patients classified by histological subtypes (cutaneous N=85 vs. occult N=11 vs. ocular N=1, mucosal N=3). **I.** *KPNA2* **J.** *DTL* **K.** *BACE2* **L.** *DTYMK*. **M-P.** Scatter dot plots showing the absolute cfRNA copies of **M.** *KPNA2* **N.** *DTL* **O.** *BACE2* **P.** *DTYMK* according to M stage (AJCC 8th edition, M0: N= 17, M1a: N=6, M1b: N=15, M1c: N=41, M1d: N=21). Significance was assessed by Kruskal-Wallis test with Dunn’s correction for multiple comparisons. Box and whisker plots represent median values and interquartile ranges. Scatter dot plots show the mean ± SEM.

**Supplementary Figure 7: A.** Longitudinal assessment of cfRNA copies of *KPNA2*, *DTL*, *BACE2*, *DTYMK* in patients with no evidence of disease (NED) at baseline. **B.** cfRNA copies of *KPNA2*, *BACE2*, *DTL*, and *DTYMK* at baseline vs. at the time of relapse were plotted for patients 7, 14, 37, and 39. Paired time points were pooled and compared by the Wilcoxon-signed rank test.

**Supplementary Figure 8: Changes in cfRNA levels during therapy classified by different therapies.**

Changes in mean (copies/mL) of cfRNA levels of **A/E.** *KPNA2* **B/F.** *DTL* **C/G.** *BACE2* **D/H.** *DTYMK* in plasma samples of melanoma patients classified as responders (N=38) and non-responders (N = 43) according to radiologic response results. The relative change in cfRNA levels at different follow-up (FU) time points (FU1: week 3-6, FU2: week 9-12; FU3: week 15-18; FU4: week > 24) were calculated in comparison to baseline (BL) (baseline time point: week 0, or up to 6 weeks before therapy start) or preceding FU evaluation. **A-D**. immune checkpoint inhibitors (ICI) (responders N=24; non-responders N=31) and **E-H**. targeted therapy (TT) (responders N=14; non-responders N=12). The data represent mean ± SEM. Asterisks indicate significance of FU time point to baseline, whereas plus signs indicate significance of the same FU time point between responders and non-responders. P values for comparison of each FU time point to baseline were determined by Two-Way ANOVA with Holm Sidak correction test. P values for comparison of paired time points between responders and non-responders were determined by t-test.

**Supplementary Figure 9: Validation of therapy predictive and prognostic relevance of KPNA2, DTL, BACE2, and DTYMK.**

**A-D**. Nivolumab treated tumors with increasing gene expression of *KPNA2*, *DTL*, *BACE2*, and *DTYMK* are associated with progression. Scatter dot plots showing the fold change in expression of **A**. *KPNA2*, **B**. *DTL*, **C**. *BACE2*, and **D**. *DTYMK* in nivolumab treated tumors (on-treatment vs. baseline) in the Riaz *et al.* dataset ^2^. P values are from Kruskal-Wallis test with Dunn’s correction for multiple comparisons test. Scatter dot plots show the mean ± SEM. RECIST classifications: CR=complete response, PR=partial response, SD=stable disease, PD=progressive disease. **E-H**. Kaplan Meier plots of overall survival of nivolumab treated melanoma patients (N=36) in the Riaz *et al.* dataset ^2^ according to on-treatment tumor gene expression profiles of **E.** *KPNA2* **F.** *DTL* **G.** *BACE2* **H.** *DTYMK*. Categorization into “low” vs. “high” was based on X-tile software ^3^. The hazard ratio is indicated for the “high” category. P values were determined by the log-rank test.

References

1 Moher D, Hopewell S, Schulz KF, Montori V, Gøtzsche PC, Devereaux PJ *et al.* CONSORT 2010 explanation and elaboration: updated guidelines for reporting parallel group randomised trials. *BMJ* 2010; **340**. doi:10.1136/BMJ.C869.

2 Riaz N, Havel JJ, Makarov V, Desrichard A, Urba WJ, Sims JS *et al.* Tumor and Microenvironment Evolution during Immunotherapy with Nivolumab. *Cell* 2017; **171**: 934-949.e15.

3 Camp RL, Dolled-Filhart M, Rimm DL. X-tile: a new bio-informatics tool for biomarker assessment and outcome-based cut-point optimization. *Clin Cancer Res* 2004; **10**: 7252–7259.
